# Role of National Policy in Improving Health Care Waste Management in Nigeria

**DOI:** 10.5696/2156-9614-8.19.180913

**Published:** 2018-09-10

**Authors:** Idoteyin Ezirim, Francis Agbo

**Affiliations:** National Agency for the Control of AIDS, Abuja, Nigeria

**Keywords:** health care waste, waste management, waste management policy, waste segregation, environmental audit

## Abstract

**Background.:**

As Nigeria strives to improve health services nationwide, there is a corresponding increase in laboratory testing, care and treatment activities, producing more healthcare waste that must be safely managed. In the past, Nigeria lacked an enabling environment for healthcare waste management, as it did not have a national health care waste management policy. However, in 2013 a policy and strategic plan for healthcare waste management was developed to address this problem.

**Objectives.:**

The present study performed an environmental safeguard audit to determine the level of implementation of the 2013 national policy in the 36 states and Federal Capital Territory in Nigeria. We also sought to determine whether the 2013 national policy has had an impact on healthcare waste management.

**Methods.:**

The present study was conducted in 1921 health facilities, selected using the probability proportional to size sampling method.

**Results.:**

The present study found that 44.8% of health facilities surveyed had healthcare waste management work plans adapted from the 2013 national policy. In addition, 89.2% of health facilities segregated waste. This is an important improvement, as previous studies reported that there was little to no waste segregation at health facilities. Furthermore, 41.4% of health facilities had designated persons or units handling healthcare waste, in contrast to previous studies which found no designated person or unit responsible for healthcare waste. However, the quality of healthcare waste management varied across states and health facilities.

**Discussion.:**

Following the introduction of healthcare waste management policy, health facilities in Nigeria have improved waste management practices. However, training, availability of required tools and functional governance structures are essential to the implementation of an effective healthcare waste management policy.

**Conclusions.:**

The study findings show that safe healthcare waste management can be implemented if the government leads by providing policy and required resources, while health facilities put standard operating procedures in place to guide day to day healthcare waste management operations.

**Participant Consent.:**

Obtained

**Ethical Approval.:**

The protocol was approved by the National Health Research Ethics Committee of Nigeria.

**Competing Interests.:**

The authors declare no competing financial interests.

## Introduction

Healthcare waste can cause disease and injury if it contains infectious waste, sharps, harmful chemical waste or radioactive waste.^1^,^ 2^ Poor management of healthcare waste can thus endanger healthcare workers and the public. Improper disposal of these wastes could also lead to environmental problems.^3^ The World Health Organization (WHO) defines healthcare waste as all waste produced from healthcare activities. Management of healthcare waste involves waste segregation, collection, storage, treatment, transportation, safe disposal and monitoring of these activities.^4^ In 2014, Ebola outbreaks in Nigeria claimed the life of 8 people and led to the WHO recommendation that proper disposal of health waste, in addition to other measures are needed to protect health workers, patients and care givers from contacting Ebola virus in health facilities.^5^,^6^ Similarly, Lassa virus, which has been problematic in Nigeria, poses a threat to healthcare workers and the general public if waste generated from infected persons is handled improperly.^7^

Nigeria has policies and regulations that protect public health and the environment, including a national policy on the environment as well as a national environmental sanitation policy.^8^,^9^ Nigeria is also a signatory to an international treaty called the Basel Convention which regulates the movements of hazardous waste between nations.^10^ According to WHO guidelines, the development of a national policy for managing healthcare waste is the first step that a government should take in addressing its management.^4^

There are several studies on waste management practices in health facilities in Nigeria. According to the results from the 2004 baseline assessment of injection safety and healthcare waste management conducted by the Making Medical Injections Safe (MMIS) project in Nigeria, 0% of hospitals used color coded bins for waste segregation.^11^ In another study on healthcare waste management in Jos, Nigeria, it was reported that waste segregation was not performed in any of the surveyed health facilities.^3^ In a similar study in Lagos, Nigeria, only 16.9% of health facilities segregated waste.^12^ Another previous study in Nigeria found that healthcare waste disposal was carried out by open air burning or burying at the facility site without prior treatment.^13^ Furthermore, in a study at a tertiary facility in Nigeria, researchers found that no designated person or unit was responsible for healthcare waste management, indicating that responsibility for it was not clearly defined.^13^

Before the existence of a national policy on healthcare waste management, the United States Agency for International Development's (USAID)/President's Emergency Plan for AIDS Relief (PEPFAR) implemented the MMIS project in Nigeria over five year period from 2004–2009.^14^ During this five-year period, the MMIS project distributed seven million safe injection commodities such as color-coded containers, sharp containers etc. worth over 104 million Naira (USD 700,000) and trained over 10,000 health workers in Nigeria on injection safety.^15^ A study by Sowande et al. in 2014 reported that during implementation of the MMIS project in Nigeria, although government support for healthcare waste management was low because Nigeria lacked a national management policy, waste management practices improved in health facilities in six MMIS project supported states (i.e. Edo, Anambra, the Federal Capital Territory, Kano, Cross River, and Lagos States).^11^ However, proper management of healthcare waste only occurred in states that had healthcare waste management projects, and remained unsatisfactory in other states in Nigeria.^11^

In 2007, the Federal Ministry of Health and Federal Ministry of Environment collaborated with the MMIS project and other partners to develop the national injection safety policy and the draft national healthcare waste management policy.^11^,^16^ Although the national injection safety policy was developed in 2007, the national healthcare waste management policy remained in draft form due to long bureaucratic delays in approval and was finally approved in September 2013.^11^ Following approval, the national healthcare waste management policy 2013 became available for implementation. To operationalize the 2013 national healthcare waste management policy, the national healthcare waste management strategic plan of 2013 – 2017 and the national healthcare waste management guidelines of 2013 were developed.^17^ The aim of the 2013 national healthcare waste management policy was to establish good waste management practices across health facilities in Nigeria.^17^,^18^ The 2013 national healthcare waste management policy also outlined how the government aimed to manage waste generated in health facilities in order to protect the public and the environment.^17^,^18^

**Abbreviations**
*MMIS*: Making Medical Injections Safer

Since the development of the 2013 national healthcare waste management policy, there seems to have been no attempt to assess its impact on healthcare waste management. However, resources have been spent on its implementation. USAID/PEPFAR spent over $2 million dollars in eight states through the AIDSFree Project in Nigeria from 2015 - 2016 to address weaknesses in healthcare waste management.^19^,^20^AIDSFree trained 112 master trainers in healthcare waste management who in turn trained a total of 13,673 health workers and waste handlers.^19^ AIDSFree also assisted facility staff to develop health care waste management plans as well as provided health care waste management supplies such as bins and liners, protective equipment for waste handlers, and sharps safety boxes to help health facilities initiate appropriate health care waste management activities.^19^ Similarly, in 2015, the Institute of Human Virology Nigeria (IHVN) trained around 40 doctors and nurses from health facilities in ten states on healthcare waste management and distributed incinerators in 19 tertiary health facilities in the country.^21^,^22^ The Federal Government of Nigeria also provided high-temperature incinerators at tertiary health facilities.^23^ The World Bank second HIV/AIDS Program Development Project (HPDP2) 2013–2015 conducted routine visits to World Bank-supported health facilities in all 36 states in Nigeria to monitor and supervise waste management processes.^24^

Given the level of investment in healthcare waste management, an environmental safeguard audit was done in 2015 by the National Agency for the Control of AIDS, Nigeria (NACA) through the World Bank second HIV/AIDS Program Development Project to assess the level of implementation of the national healthcare waste policies and guidelines in the 36 states and Federal Capital Territory.

## Methods

The study was conducted in 2015 using quantitative and qualitative methods for data collection.^17^ The qualitative study was conducted in six states (one state per geopolitical zone). In each state, the key informants were staff from state Ministry of Health and safeguard officer in State Agency for the Control of AIDS (SACA) in Nigeria. In total, 12 key informants were interviewed.

The quantitative study was conducted in health facilities in all Nigerian states. Altogether, 1,921 public and private health facilities were included in the study. The probability proportional to size (PPS) sampling procedure was used to select 1,921 health facilities from 7,667 health facilities that provided HIV/AIDS services in the country.^17^,^25^ The sampling was done within a 95% confidence interval, 5% margin of error and 10% inflation due to non-response or dropout. Sampling was done in two stages. In the first stage, health facilities in each state were stratified by the local government (i.e. districts), and in the second stratification, health facilities were stratified into tertiary, secondary and primary health facilities.^17^ There are three levels of health facilities in Nigeria: primary, secondary and tertiary. Each health facility level is supported by an administrative level of government. The local government is responsible for the primary health centers, the state government is responsible for secondary health centers and the federal government is responsible for tertiary health centers. The stratification of health facilities was done to ensure that the study sample was representative.

### Ethics statement

The protocol was approved by the National Health Research Ethics Committee of Nigeria (NHREC). All participants who agreed to take part in the study were required to sign the informed consent before participating.

### Data collection

The qualitative study involved the review of published records on healthcare waste management and interview of key informants using a key informant guide. The published records reviewed included healthcare waste management policy, healthcare waste management strategic plan, and reports from various projects providing healthcare waste management interventions, etc.

During the quantitative study, a checklist was administered to health workers at health facilities. The checklist for this study was adapted from an environmental and social safeguard questionnaire published online by the World Bank.^26^ The checklist included sections on healthcare waste (its identification, characterization and segregation at the health facility), emergency preparedness and response, safety, health and environment, as well as a section on the use of an incinerator for waste disposal.

The checklist was pretested before field work and feedback from the pretest was used to revise the checklist. Data collectors who had participated in similar surveys in the past were used in the present study. In addition, all data collectors were trained on how to use the checklist to ensure they had a common understanding of the checklist. Field supervisors reviewed completed checklists and conducted spot checks on data collectors.

### Data analysis

Data from surveyed health facilities were entered into IBM Statistical Package for the Social Sciences 22.0 (SPSS 22.0) software. Data were then cleaned, edited and analyzed in SPSS.

## Results

**Figure 1:**
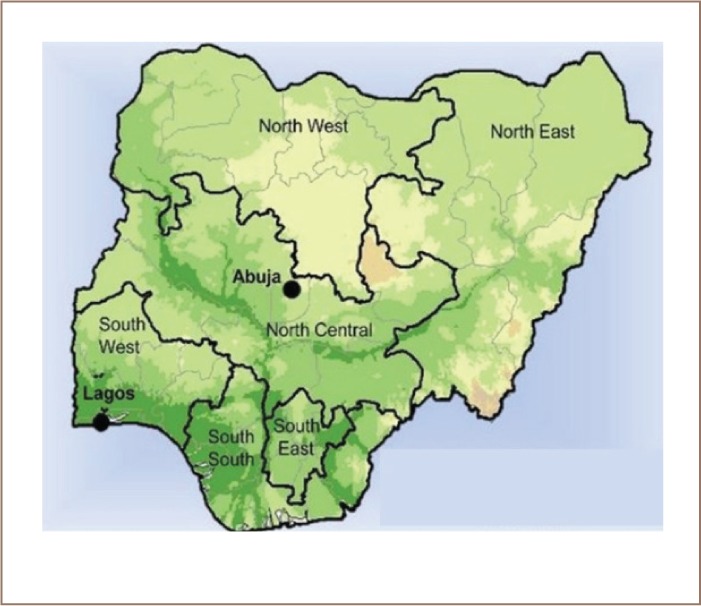
Map of Nigeria showing the six geopolitical zones

Out of 1,921 health facilities in the study, 64% were government-owned primary healthcare facilities, 12.4% were government-owned secondary health care facilities, 1.6% were government-owned tertiary facilities and 22% were private health facilities. The health facilities were spread across the six geopolitical zones of Nigeria and covered all Nigerian states *(Table 1, Figure 1)*.

**Table 1:** Distribution of Health Facilities by Geopolitical Zones

**Geopolitical Zone in Nigeria**	**Number of Health Facilities Studied**
***North East***	121
***North West***	171
***North Central***	426
***South East***	451
***South West***	226
***South South***	526
***TOTAL***	1,921

The study findings revealed that 44.8% of all facilities sampled had healthcare waste management plans developed from the national healthcare waste management policy *(Table 2).* In addition, 41.4% of all health facilities had a designated person or department with the responsibility and resources to operate and monitor healthcare waste disposal on a daily basis *(Table 2).* Waste segregation procedures at health facilities were observed during the study. The findings indicated that 80.4% of health facilities were familiar with recommended methods for waste segregation in the national healthcare waste policy. In addition, color-coded containers for segregating biological wastes and sharps were seen at health facilities *(Table 2).* Waste segregation was carried out in 89.2% of health facilities *(Table 2).*

**Table 2:** Waste Management Practices at Health Facilities in Nigeria

**Health Facility**	**Number of Health Facilities Studied**	**Percentage of Health Facilities with Waste Management Work Plans**	**Percentage of Health Facilities with Designated Person/Office Responsible for Waste Management**	**Percentage of Health Facilities that Dispose of Infectious Waste Properly**	**Percentage of Health Facilities that Segregate Waste**	**Percentage of Health Facilities with Biohazard and Sharp Containers**
Type of Facility by Level of Care						
***Tertiary***	31	79.3%	82.8%	90.0%	96.7%	100.0%
***Secondary***	238	51.4%	57.2%	71.8%	87.2%	91.0%
***Primary***	1230	42.9%	37.7%	72.4%	89.0%	87.8%
***Private***	422	44.2%	40.5%	73.2%	90.4%	89.0%
***TOTAL***	1,921	44.8%	41.4%	72.7%	89.2%	88.7%

The results of the present study showed a reoccurring pattern in that tertiary facilities had better waste management practices than secondary and primary health facilities. In the present study, 79.3% of tertiary health facilities, 51.4% of secondary health facilities and 42.9% of primary health facilities had waste management plans *(Table 2).*

**Figure 2:**
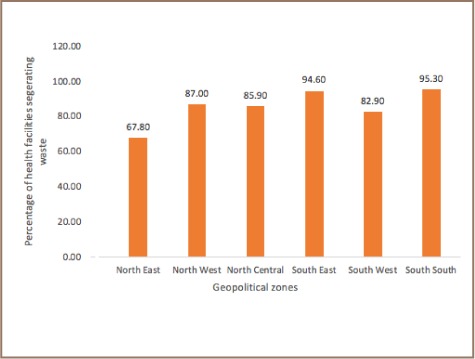
Percentage of health facilities segregating waste across geopolitical zones in Nigeria.

The implementation of the national healthcare waste management policy varied across the country. The present study found that the percentage of health facilities practicing waste segregation was highest (95.3%) in the South South zone and lowest (67.8%) in the North East zone in Nigeria *(Figure 1)*. Comparing hospitals segregating waste with the availability of biohazard and sharp containers for waste segregation, the results showed that although the North East zone had the most facilities (95.7%) with biohazard and sharp containers, waste segregation was lowest in this zone *(Figure 2)*. Meanwhile, the South South zone, where 92.6% of health facilities had biohazard and sharp containers, had the highest number of facilities segregating waste *(Figure 2)*. Healthcare waste management plans developed from the national policy were found in 62.7% of health facilities in the South South zone of Nigeria, while in the North East zone, 16.1% of health facilities had healthcare waste management plans.

In the present study, 22.2% of health facilities had standard incinerators for proper treatment of hazardous waste *(Table 3)*, while the rest disposed of waste by open air burning or burying at the facility. In 68.3% of health facilities, health workers handling waste used personal protective equipment (PPE) *(Table 3).* However, there were no waste management committees in any health facility in the present study.

**Figure 3:**
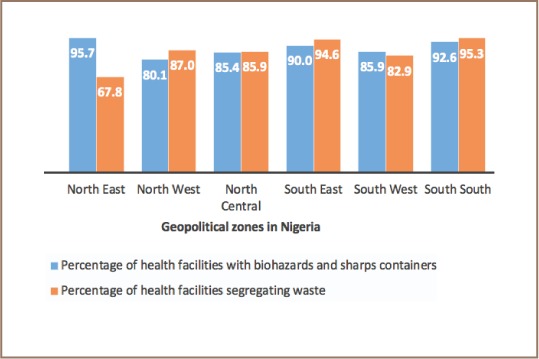
Percentage of health facilities with biohazard and sharp containers segregating waste across geopolitical zones in Nigeria.

**Table 3:** Waste Management Practices at Health Facilities in Nigeria

**Health Facility**	**Number of Health Facilities Studied**	**Percentage of Health Facilities with Incinerators**	**Percentage of Health Facilities that Use PPE**
Type of Facility by Level of Care			
***Tertiary***	31	76.7%	90.0%
***Secondary***	238	36.5%	75.0%
***Primary***	1230	19.5%	63.6%
***Private***	422	18.1%	76.8%
***TOTAL***	1,921	22.2%	68.3%

**Abbreviations**: PPE, personal protective equipment

## Discussion

Previous studies in Nigeria reported 0% and 16.9% waste segregation in health facilities.^3^,^11^,^12^ The current study found that 89.2% of health facilities segregated waste at the point of generation, which is a substantial improvement. A review article of healthcare waste management in developing countries found that training of medical staff improved some aspects of waste management, but did not address the cost of materials for waste management (e.g. containers, incinerators) and infrastructure (e.g. administrative structure, regulations).^27^ In addition, the review paper concluded that a mix of training programs, administrative and governance solutions were required to tackle waste management in health facilities.^27^ Similarly, in a health facility in Malaysia, despite provision of policy and guidelines on waste management and training of health facility staff, poor compliance with waste segregation continued. Further investigation showed that health workers at a Malaysian hospital were not segregating waste because the tools required for waste segregation were unavailable.^28^ Improvement in waste segregation in Nigeria occurred because after healthcare waste policy rollout, health workers were trained in waste management, health facility waste management plans were developed, tools for segregation, transportation and disposal of waste were provided and staff responsible for waste management were identified. The national healthcare waste management policy will have had little or no impact on waste management in Nigeria if all these processes are not implemented. Although there were no repercussions like fines or fees for facilities if they did not implement the healthcare waste management policy, it is possible that regular monitoring and supervision of facility healthcare waste practices through monitoring visits to these facilities may have contributed to compliance.^24^

In the present study, health facilities in the North East and South South zones were provided with biohazard and sharp containers (95.7% and 92.6%, respectively), but more health facilities in the South South zones (62.7%) had waste management plans than health facilities in the North East zone (16.1%). This provides insight into why waste segregation was high in the South South and low in the North East geopolitical zones of the country despite the availability of biohazard and sharp containers in both zones. According to a study by Almuneef and Memish, healthcare waste generation reduced at their hospital after a few months of introducing the waste management policy and implementing a facility waste management plan.^29^ This suggests that in addition to providing biohazard and sharp containers, it is essential for health facilities to develop waste management plans to effectively manage healthcare waste.

Comparing the availability of health facility waste management work plans in the present study with previous studies, it was found that in previous studies, health facilities lacked healthcare waste management work plans, whereas in the present study, 44.8% of health facilities had health facility healthcare waste management work plans.^13^,^12^ Despite this improvement, it is important that the Ministry of Health, in collaboration with its partners, ensure that health facilities in all states in Nigeria develop waste management plans.

Nigeria has progressed from having no designated person or unit accountable and responsible for healthcare waste in hospitals, to the current situation, where 41.4% of health facilities have designated persons or units handling healthcare waste.^13^ According to WHO guidelines, a designated person or team should have overall responsibility for overseeing the day to day operation and monitoring of a facility's waste management plan.^4^ The designated person or unit essentially coordinates the activities of diverse hospital staff in various departments involved in waste management. In the present study, health facilities with a designated person or team responsible for handling health waste demonstrated better waste management practices.

In Nigeria, previous studies have reported that healthcare waste disposal was carried out by open air burning or burying at the facility site without prior treatment.^13^ The present study found that currently, 22.2% of health facilities use incinerators for waste disposal. However, incinerators were primarily available in tertiary health facilities, while open air burning and burying of waste is still performed in secondary and primary health facilities. Therefore, more needs to be done to increase the availability of incinerators at a greater number of health facilities.

## Conclusions

Prior to the development of the 2013 healthcare waste management policy, studies identified the lack of a national healthcare waste management policy in Nigeria as an important barrier to proper healthcare waste management.^11^,^12^ The findings of the present study confirm that implementation of the 2013 national healthcare waste management policy has improved waste management practices in Nigeria. Furthermore, the development of a healthcare waste management policy demonstrates government leadership and commitment to waste management. Thus, it is possible to implement safe healthcare waste management if the government leads the effort by providing policies and resources to facilitate the implementation of standard operating procedures in health facilities to guide day to day healthcare waste management operations.

Finally, the present study reported that healthcare waste management practices varied across facilities, but was not able to determine the reasons for the better quality of waste management observed in tertiary hospitals compared to secondary, primary and private health facilities. The aim of this study was to determine if implementation of healthcare waste policy improved waste management in health facilities, but further studies are needed to explain these improvements. Another limitation of the present study was that all health facilities selected for this study provided HIV/AIDS services and facilities which did not provide HIV/AIDS services were excluded.
